# New Insights into the Connections between Flooding/Hypoxia Response and Plant Defenses against Pathogens

**DOI:** 10.3390/plants13162176

**Published:** 2024-08-06

**Authors:** Pablo García, Shreenivas Singh, Emmanuelle Graciet

**Affiliations:** 1 Department of Biology, Maynooth University, W23 X021 Maynooth, Co. Kildare, Ireland; pablo.garciagonzalezdeheredia.2022@mumail.ie (P.G.); shreenivas.singh@mu.ie (S.S.); 2 Kathleen Lonsdale Institute for Human Health Research, Maynooth University, W23 X021 Maynooth, Co. Kildare, Ireland

**Keywords:** flooding, hypoxia, plant immunity, plant defenses against pathogens, combined stress

## Abstract

The impact of global climate change has highlighted the need for a better understanding of how plants respond to multiple simultaneous or sequential stresses, not only to gain fundamental knowledge of how plants integrate signals and mount a coordinated response to stresses but also for applications to improve crop resilience to environmental stresses. In recent years, there has been a stronger emphasis on understanding how plants integrate stresses and the molecular mechanisms underlying the crosstalk between the signaling pathways and transcriptional programs that underpin plant responses to multiple stresses. The combination of flooding (or resulting hypoxic stress) with pathogen infection is particularly relevant due to the frequent co-occurrence of both stresses in nature. This review focuses on (i) experimental approaches and challenges associated with the study of combined and sequential flooding/hypoxia and pathogen infection, (ii) how flooding (or resulting hypoxic stress) influences plant immunity and defense responses to pathogens, and (iii) how flooding contributes to shaping the soil microbiome and is linked to plants’ ability to fight pathogen infection.

## 1. Introduction

Plants live in a complex and ever-changing environment, which can be a source of abiotic or biotic stresses that adversely affect plant growth, development, and productivity. For decades, research efforts have focused on the study of physiological and molecular responses to individual stresses, yielding an in-depth understanding of the molecular and biochemical mechanisms that underpin plants’ physiological responses to specific (a)biotic stresses. However, in their natural habitat, plants frequently face combinations of sequential or simultaneous (a)biotic stresses. Notably, these stress combinations result in the deployment of acclimation strategies that are different, and sometimes even contrasting, to those triggered under each of the individual stresses (reviewed in [1,2,3,4,5,6]). In some cases, the response to combined stresses cannot be predicted from our knowledge of plants’ responses to each of the individual stresses [3,7,8,9]. Another level of complexity originates from the fact that stress responses can be influenced by both the duration and severity/amplitude of the stress [10]. In addition, plant exposure to a single stressor can trigger cross-tolerance to other (unrelated) stressors [11], thus highlighting the connections and the regulatory crosstalk that exist between signaling pathways and responses to different stresses.

In recent years, there has been a stronger interest in understanding how plants respond to two (or more) simultaneous or sequential stresses (recently reviewed in [12]). Considering that plant responses to both types of stresses share signaling components, a key question has been whether common signaling pathways contribute to integrating signals from abiotic/biotic stress combinations to trigger a coordinated response [13]. The combination of abiotic/biotic stresses can conceptually be approached from three complementary angles: (i) How does pathogen infection affect the host’s tolerance to abiotic stress(es)? (ii) How does a given abiotic stress influence the onset of innate immune responses and the activation of defenses against pathogens? (iii) How does a specific abiotic stress affect pathogen distribution and microbial communities in general?

The more frequent occurrence of floods as a consequence of global climate change [14] has become an important concern because of their devastating effects on crop yields [15,16], with the global crop production loss due to flooding already estimated to have been USD 5.5 billion for the period between 1982 and 2016 [17]. Flooding can result in plant submergence (plants being fully underwater) or waterlogging (when only the soil is saturated with water and the aerial parts of the plants are unaffected) [18]. In both cases, the replacement of air with water results in decreased oxygen availability for the plant (hypoxia) or even the absence of oxygen (anoxia). The excess of water in the soil triggers additional physico-chemical changes, such as a decrease in the nitrogen sources absorbed by plants or metal toxicity [19]. Several studies have shown how pathogen infection often occurs sequentially to or simultaneously alongside flooding in natural or agricultural settings, making it particularly relevant to determine how plant immunity and defenses against pathogens can be influenced by flooding and the reduced oxygen availability associated [12,20,21,22,23]. Here, we review advances in our understanding of how flooding and/or hypoxia contribute to shaping plant immunity and defenses against pathogens, including considerations on how to experimentally approach such biological questions. This review will also extend to the effects of flooding on the composition of the plant microbiome and the rhizosphere and how these changes contribute to either pathogen infection or plant protection.

## 2. Experimental Strategies to Study the Crosstalk between Flooding and Plant Defense

The experimental design of combined stress experiments can be complex, as stress may be applied simultaneously or sequentially, with the duration of the recovery period between each stress also affecting the outcome of sequential treatments [24]. In the case of combined flooding and pathogen infection experiments (noted as flooding_x_pathogen), a wide range of experimental approaches may be used for each of the stresses applied. In the case of flooding, plants may be treated with either waterlogging or submergence. These two treatments differ in that in the case of waterlogging, only the roots will experience hypoxia, while the leaves may receive mobile/systemic signals from the waterlogged roots. During submergence, the entire plant will experience hypoxia. The use of waterlogging/submergence also encompasses other stresses associated with flooding, such as a reduced nitrogen availability or metal toxicity. The effects of such treatments depend on the soil types, which can make it difficult to reproduce the results in different labs [25]. An alternative is the use of hypoxia chambers, e.g., gas replacement using either chemical methods or flushing with nitrogen or argon (with the principle of each method, as well as the pros and cons, reviewed in [25]), to decrease the oxygen levels. Irrespective of whether waterlogging, submergence, or hypoxia treatments are applied, the duration of the treatment will influence the outcome of the experiment, as well as whether it is combined with dark conditions (to mimic the muddy water conditions that accompany submergence and/or to enhance hypoxic stress by limiting oxygen production via photosynthesis). For example, prolonged submergence treatment in the dark will result in the induction of a starvation response due to the inhibition of photosynthesis [26].

Similarly, the plant immune and defense responses may be studied using different approaches (in combination or sequentially with flooding/hypoxia), such as eliciting innate immune responses specifically by treating plants with purified pathogen-associated molecular patterns (PAMPs). For example, the 22-amino-acid peptide flg22 from the N-terminus of bacterial flagellin may be used as a model PAMP to elicit pattern-triggered immunity (PTI) [27]. Alternatively, pathogen inoculation may be used to assess the crosstalk between flooding/hypoxia and plant defenses. Pathogens of different kingdoms (e.g., bacteria or fungi) and with different lifestyles (i.e., biotrophs, necrotrophs, or hemi-biotrophs) can have potentially different outcomes, as the plant defense responses will differ, especially as a function of the pathogen’s lifestyle. For example, defense against biotrophic pathogens that retrieve nutrients from living tissue typically relies on salicylic acid (SA) signaling, whereas plant responses to necrotrophic pathogens (which trigger cell death) relies on jasmonic acid (JA) and ethylene signaling (reviewed in [28]).

The combination of flooding_x_pathogen has so far been more commonly applied than hypoxia_x_pathogens or hypoxia_x_PAMP, likely because it more closely mimics the natural environment and may be more directly linked to applications (e.g., [21,29,30,31]). However, when foliar pathogens are used (as opposed to soilborne pathogens), the inoculation is often performed after the flooding treatment, during a reoxygenation phase, thus making it difficult to draw clear and generalized conclusions (e.g., [29]). The use of hypoxia_x_PAMP treatments offers advantages in that the experimental conditions may be more tightly controlled and specific aspects of plant innate immunity may be dissected after abstraction from the compound nature of flooding and the more complex “molecular conversation” between the pathogen and its host [30,31]. In this case, hypoxia and PAMP treatments can also be simultaneous.

Genes involved in regulating the crosstalk between two (or more) stresses are often first identified because of their role(s) in plant responses to more than one stress. Such dual/multifactorial roles can be identified by comparing stress-specific transcriptomic datasets to identify genes that are differentially expressed in response to more than one stress [32]. Subsequent analysis of mutants for such genes under different individual stresses contributes to confirming a dual/multifactorial role in plant responses to multiple stresses. While some of these genes may be general stress response genes, involved in conserved signaling pathways, others may play more specific roles. Experiments with simultaneous stresses can further reveal roles of such genes in the integration of information from multiple stresses or in the regulation of the crosstalk between different stress response pathways. For example, Hsu et al. have shown that the submergence of 9-day-old *Arabidopsis thaliana* seedlings resulted in the transcriptional regulation of a number of genes known to play roles in plant defense, such as several *PATHOGENESIS-RELATED* (*PR*) genes, *FLG22-INDUCED RECEPTOR-LIKE KINASE 1* (*FRK1*), and WRKY family transcription factors, which are well known for their roles in plant defense [29]. They subsequently confirmed the up-regulation of *WRKY22* upon submergence and found that two mutant alleles of *WRKY22* also displayed increased sensitivity to the model bacterial pathogen *Pseudomonas syringae* DC3000 when inoculated after a submergence pre-treatment. Other aspects of plant responses to flooding/hypoxia, pathogens/PAMP, or combined treatment can also be monitored, with the activity of common signal transduction pathways, gene expression differences, or the accumulation of marker proteins for each of the stresses potentially of interest. These are discussed in more detail and with examples in the sections below.

## 3. Common Signal Transduction Pathways between Flooding/Hypoxia Response and Plant Innate Immunity/Defense

### 3.1. Pathogen and Hypoxia Sensing

**Figure 1:**
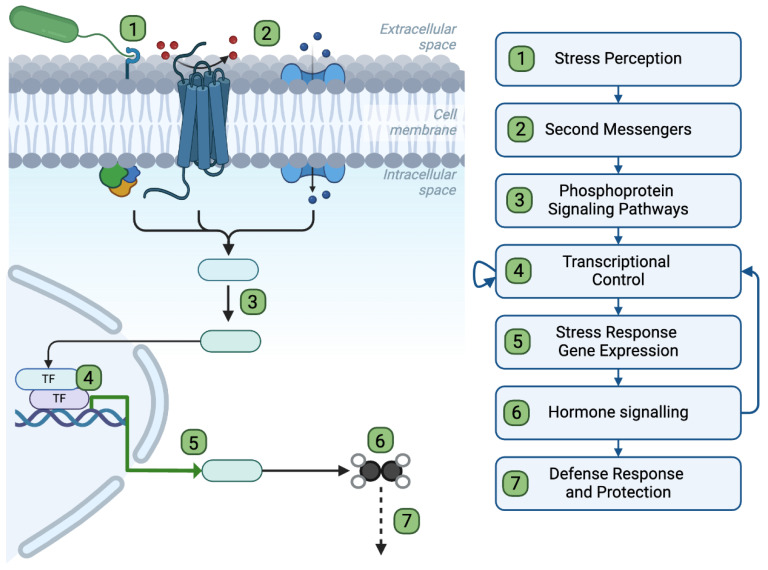
Overview of signaling events downstream of PAMP perception. Pathogens are detected via either PAMPs or pathogen effectors. PAMPs bind to and activate PRRs at the plant cell surface (1). This leads to the generation of second messengers such as Ca^2+^ (blue circles) or ROS (red circles) (2). These second messengers contribute to initiating phosphorylation cascades that result in the activation of transcription factors (3) which trigger the expression of defense genes (4 and 5). This transcriptional reprogramming results in the activation of the defense response and of hormone signaling pathways (6–7) that further contribute to plant defenses against pathogens. Steps (1) to (6) also apply to the perception of and response to abiotic stresses.

**Figure 2:**
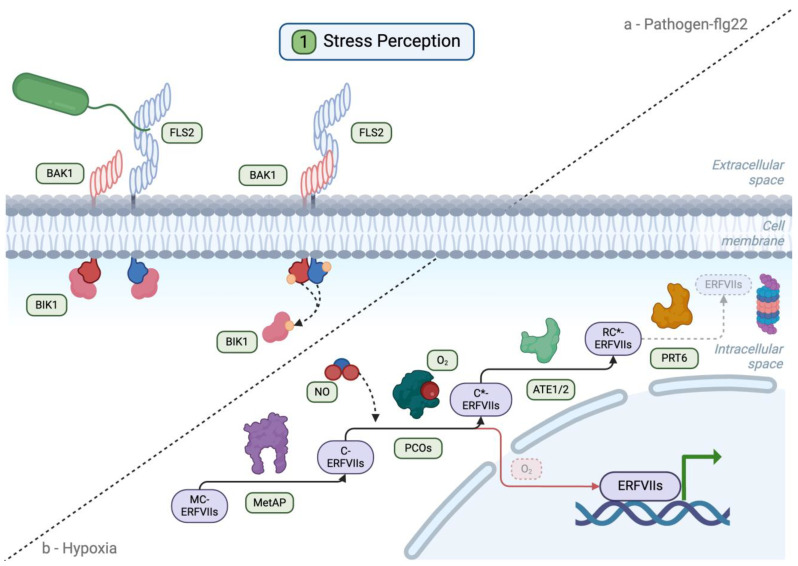
Hypoxia and flg22 stress sensing. (**a**) In its non-activated state, FLS2 interacts with BIK1 and cycles between the plasma membrane (PM) and endosomal compartments. Upon flg22 binding, FLS2 forms a stable complex with BAK1 stimulating phosphorylation (orange circles), which, in turn, triggers the release of BIK1 and the production of second messengers. (**b**) Under normal oxygen (O_2_) levels, the N-degron pathway targets ERF-VII transcription factors for degradation through the action of methionine amino peptidases (MAPs), which remove the initial methionine (M) of ERF-VII transcription factors; plant cysteine oxidases (PCOs), which oxidize the newly exposed N-terminal cysteine (C* denotes oxidized cysteine); arginyl transferases (ATE1 and ATE2) that conjugate arginine (R) to the N-terminus; and the E3 ubiquitin ligase PRT6, leading to the polyubiquitination of the ERF-VII transcription factors and their degradation by the 26 proteasome. Under low oxygen levels (red dashed square and arrow), the N-degron pathway is inhibited at the PCO level, thus enabling the stabilization of the ERF-VII transcription factors, their accumulation in the nucleus, and the activation of hypoxia response genes. NO is known to stabilize ERF-VII transcription factors through an as-of-yet unknown mechanism involving PCOs.

Plants detect pathogens via different types of receptors, including (i) transmembrane receptor-like kinase PRRs, which bind PAMPs (Figure 1) and activate PTI, and (ii) intracellular nucleotide-binding site leucine-rich repeat (NBS-LRR or NLR) receptors that initiate effector-triggered immunity (ETI) following the (in)direct recognition of specific pathogen effectors (i.e., molecules or proteins whose activity contributes to pathogen virulence in the absence of detection by cognate NLRs) (reviewed in detail in [33,34,35,36]). For example, the model PAMP flg22 is bound by the FLAGELLIN-SENSITIVE2 (FLS2) PRR [37,38,39], leading to the formation of an immune receptor complex at the plasma membrane through the interaction of FLS2 with BRI1-ASSOCIATED RECEPTOR KINASE 1 (BAK1) and the receptor-like cytoplasmic kinase BOTRYTIS-INDUCED KINASE 1 (BIK1) [39,40,41,42,43,44] (Figure 2). Downstream of the activation of this complex, phosphorylation-dependent signaling relays the signal from the plasma membrane to the nucleus, where genome-wide gene expression changes take place. Second messengers (such as calcium (Ca^2+^) and reactive oxygen species (ROS)), as well as plant hormone signaling pathways, also contribute to the onset of the immune and defense responses (Figure 1).

In contrast, hypoxia sensing in plants is mediated by the activity of oxygen-dependent proteins, including PLANT CYSTEINE OXIDASE (PCO) enzymes that oxidize the N-terminal cysteine residue of group VII ETHYLENE RESPONSE FACTOR (ERF-VII) transcription factors, which act as the master regulators of the hypoxia response program [45,46] (Figure 2). Under normoxic conditions, this PCO-dependent cysteine oxidation continuously targets ERF-VIIs for degradation by the ubiquitin-dependent N-degron pathway [47,48], while under hypoxia, ERF-VIIs become stabilized due to the inhibition of PCOs [49,50,51]. These transcription factors translocate to the nucleus and play a crucial role in regulating the expression of core hypoxia response genes, as well as other genes whose activity is important for plant survival to flooding/hypoxia [15,52,53,54,55,56,57]. Even though sensing of pathogens and flooding/hypoxia involves different mechanisms, these converge onto common second messengers, signaling pathways, and transcription factors (Figure 1), which could contribute to regulating the crosstalk between the responses to flooding/hypoxia and pathogens while also serving as platforms for signal integration from both stresses and the onset of an adapted response under combined stress.

### 3.2. Reactive Oxygen Species (ROS), Nitric Oxide (NO), and Calcium (Ca^2+^) Signaling

ROS are highly reactive chemical species that play a signaling role in response to a multitude of abiotic stresses (including flooding/hypoxia), as well as in response to pathogen and PAMP perception. ROS often act in conjunction with Ca^2+^ and with nitric oxide (NO), as these molecules regulate each other’s signaling pathways and can be up-regulated early after the perception of either flooding/hypoxia or pathogen/PAMPs. For example, the influx of Ca^2+^ into the cytosol peaks around 4 to 6 min after PAMP treatment (e.g., flg22 or elf18 (a PAMP peptide from bacterial ELONGATION FACTOR THERMO UNSTABLE (EF-Tu)) [58,59,60]. Cytosolic Ca^2+^ also plays a role in hypoxia and anoxia responses [61,62,63,64] and includes other sources of Ca^2+^ [65], such as vacuolar Ca^2+^ through the role of CATION EXCHANGER 2 (CAX2) [66]. The use of intracellular Ca^2+^ sensors in submerged leaf tissue has indicated that the free Ca^2+^ concentrations peak at 6 to 8 h [67]. Similarly to ROS, NO is produced within minutes of either pathogen/PAMP perception [68,69,70] or hypoxia sensing [71,72,73,74,75]. NO is also particularly important in hypoxia sensing, as it contributes to the oxidation of the N-terminal cysteine of ERF-VII transcription factors under normoxia, preventing their accumulation and the unnecessary activation of hypoxia-responsive genes [47,72]. Notably, upon hypoxia, early ethylene signaling events result in a rapid decrease in NO, allowing an early stabilization of ERF-VIIs and the activation of a hypoxia response [76].

**Figure 3:**
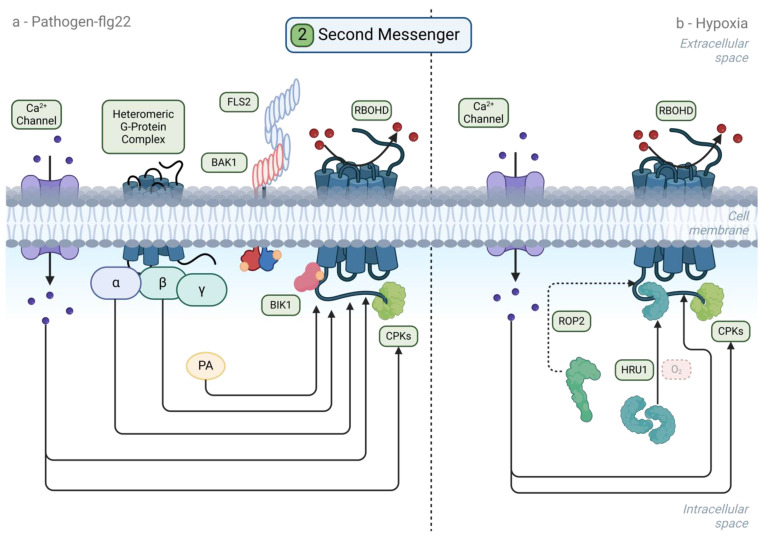
Second messengers and signaling pathways downstream of pathogen or hypoxia sensing. (**a**) After sensing flg22, phosphorylated BIK1 interacts with RBOHD, as well as with other proteins such as GPA1 or AGB1, which are alpha and beta subunitsof a G-protein complex. Small molecules such as phosphatidic acid (PA) can also interact with RBOHD, inducing its activity and producing O_2_-, which is rapidly dismutated into H_2_O_2_ in the apoplast. (**b**) HYPOXIA-RESPONSIVE UNIVERSAL STRESS PROTEIN 1 (HRU1)-HRU1 dimers are monomerized under hypoxic conditions. HRU1 migrates to the plasma membrane and interacts with RBOHD, thus contributing to its activation. ROP2 is also known to act upstream of RBOHD through an unknown mechanism. Common regulatory mechanisms of RBOHD include the direct binding of Ca^2+^ to RBOHD’s EF hands and CPKs such as CPK5, which interacts with RBOHD’s N-terminus.

The convergence of Ca^2+^, ROS, and NO signaling in regulatory events following flooding/hypoxia or pathogen/PAMP perception can be illustrated through the regulation of the ROS-producing enzyme RESPIRATORY BURST HOMOLOG D (RBOHD), a key regulator of plant responses to a wide range of stresses (reviewed in [77,78,79]), including flooding/hypoxia and pathogen/PAMP detection (Figure 3). RBOHD (and other members of the RBOH family) is a transmembrane enzyme that uses NADPH as an electron donor to produce superoxide anions in the apoplast, where they can be converted into the more stable hydrogen peroxide (H_2_O_2_) by apoplastic enzymes such as peroxidases [80] and superoxide dismutases [81]. H_2_O_2_ is membrane-permeable and can enter the cytosol via aquaporins, thus further contributing to intracellular ROS signaling [82,83,84,85].

Upon the perception of flg22, RBOHD associates with an FLS2 supercomplex that phosphorylates RBOHD, together with the receptor-like cytoplasmic kinase BIK1 [43,86] and Ca^2+^-dependent protein kinases (e.g., CPK5 [87]), induced by an intracellular Ca^2+^ burst [87,88,89]. Moreover, Ca^2+^ itself binds to RBOHD EF-hand motifs, thus activating RBOHD in conjunction with phosphorylation events [90]. The NO-dependent S-nitrosylation of both BIK1 and RBOHD (among other proteins) plays an important role in the activation of PTI [91,92], with RBOHD S-nitrosylation resulting in its repression in the context of ETI-related cell death [92]. RBOHD regulation during PTI involves additional post-translational mechanisms, including PBL13-INTERACTING RING DOMAIN E3 LIGASE (PIRE)-mediated ubiquitylation and degradation, as well as multiple phosphorylation events [93] (Figure 3).

Upon flooding/hypoxic stress, RBOHD-dependent ROS production also plays important roles in mediating plant tolerance to flooding/hypoxia [94,95,96,97,98], including systemic signals, in conjunction with Ca^2+^ signaling [99,100,101]. However, the reduced tolerance to anoxia (but not hypoxia) of the *rbohd* mutant compared to the wild type suggests that RBOHD may play a more important role under anoxia than hypoxia [97]. RBOHD activation upon flooding/hypoxia (at 0.1% oxygen) depends on Ca^2+^-dependent kinases, including CPK16 and possibly CPK28, through a mechanism that involves the phosphorylation and stabilization of RBOHD [102] (Figure 3). Nonetheless, the opposite phenotypes of the *rbohd* (more sensitive) and *cpk16* (more tolerant) mutants under oxygen <0.1% and upon submergence suggest a more complex mechanisms for the regulation of ROS upon hypoxia [102]. RBOHD also regulates Ca^2+^ influx upon hypoxia, and this function is partially redundant with that of RBOHF [98]. RBOHD regulation during flooding/hypoxia involves additional post-translational events such as its interaction with HYPOXIA-RESPONSIVE UNIVERSAL STRESS PROTEIN 1 (HRU1), a positive regulator of RBOHD and ROS production under anoxia [103], downstream of G-protein signaling [104,105]. However, connections between NO and RBOHD activity have not been established under flooded or hypoxic conditions.

Despite our understanding of the integrated ROS-, Ca^2+^- and NO-dependent biochemical mechanisms upon either flooding/hypoxia or pathogen/PAMP treatments, little is known about how they contribute to the crosstalk between these stress response pathways in the context of combined stresses (e.g., flooding_x_pathogen or hypoxia_x_PAMP). This contrasts with other abiotic stress_x_biotic stress combinations, for which some molecular mechanisms have been dissected (reviewed in [13]). Studying the role of these secondary messengers, especially ROS and Ca^2+^, under combined stress would also be particularly relevant to understanding cell-to-cell communication and systemic signaling. For example, RBOHD-produced ROS signals propagate at speeds of up to 8.4 cm/min through plant tissues in response to some (a)biotic stresses such as wounding or aphid feeding [106,107,108,109], but less is known about combined stresses [110] and in particular hypoxia/flooding combined with biotic stress.

### 3.3. Mitogen-Activated Protein Kinase (MAPK) Signaling

**Figure 4:**
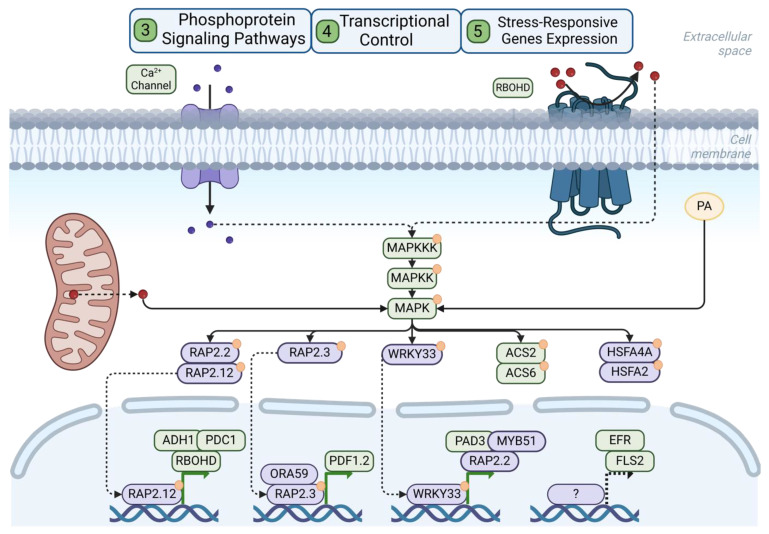
MAPK signaling and transcriptional regulation. The induction of MAPK signaling upon pathogen/PAMP or hypoxia sensing involves different mechanisms, including Ca^2+^ (purple circles) signaling, ROS (red circles), and PA. MAPK signaling triggers the phosphorylation (orange circles: phosphate groups) of different transcription factors such as RAP2.12, RAP2.3, or WRKY33, regulating their activity and sub-cellular localization. Several transcription factors that act downstream of MAPK signaling have been shown to play dual roles in pathogen defense and response to hypoxia.

MAPK signaling plays essential roles in the activation of immune/defense and hypoxia responses, with rapid activation following pathogen/PAMP or flooding/hypoxia sensing (e.g., MAPK signaling is activated within 1–2 min of flg22 perception [111]). Several events contribute to the activation of MAPK signaling, downstream of the phosphorylation (and activation) of initial MAP3K, which then phosphorylates/activates MAP2Ks. The latter finally activates MAPKs through their phosphorylation (reviewed in [112,113]). MPK3 and MPK6 play functionally redundant roles in both plant immunity (reviewed in [114]) and hypoxia response [115] and are probably the most studied MAPKs (together with MPK4) in the context of these two stress response pathways (Figure 4). Generally, *mpk3/6* double-mutant plants exhibit increased susceptibility to pathogens [116,117] and submergence [118]. Downstream of MAPK activation, additional MPK3/6-dependent phosphorylation events take place. For example, upon PAMP/pathogen perception, MPK3/6 phosphorylate and activate transcription factors such as WRKY33 [116,119], a well-known regulator of plant defenses against pathogens (see below), as well as the ERF-VII transcription factor RAP2.3 (in response to *B. cinerea*) [120], which was also identified as a substrate of MPK3/6 in an in vitro study [121]. The phosphorylation of RAP2.3 by MPK3/6 in response to a pathogen suggests a link between immunity and hypoxia response via MAPK signaling. Flooding/hypoxia also activates MPK3/6 [115,122], which then phosphorylate the ERF-VII transcription factor RAP2.3 [118]. In addition, the activation of MPK3/6 upon hypoxia was shown to involve the phospholipase D-dependent release of phosphatidic acid [118], as well as ROS production [115]. While all of the regulatory mechanisms discussed above were identified using the model plant *Arabidopsis thaliana*, experiments in rice (*Oryza sativa*) have confirmed the activation of MPK3 during submergence, but this time via a mechanism involving the *SUB1A1* allele of the *SUB1A* gene, an ERF-VII transcription factor. This allele is specifically associated with flooding tolerance in rice and has been shown to be phosphorylated by MPK3 upon submergence while also activating the expression of *MPK3*, thus establishing a positive feedback loop between these two proteins [123] (Figure 4).

While either pathogen or flooding treatments appear to involve the phosphorylation and activation of ERF-VII transcription factors by MPK3/6, as well as positive regulation of *MPK3/6* expression by ERF-VII transcription factors, simultaneous treatment with hypoxia and flg22 does not affect the transcriptional regulation of *MPK3/6* while resulting in the decreased phosphorylation of both MPK3/6 compared to treatment with flg22 alone. Interestingly, this negative effect of simultaneous hypoxia_x_flg22 treatment appears to be ERF-VII-independent [30]. It is unclear, though, whether a similar result would be obtained if plants were simultaneously treated with submergence and a pathogen.

### 3.4. The Dual Role of Transcription Factors in the Response to Flooding/Hypoxia and Pathogen/PAMPs

Statistically significant overlaps have been identified between the transcriptional response of Arabidopsis plants to either flooding/hypoxia or pathogens/PAMPs [29,30,31]. For example, Hsu et al. have shown that submergence of 9-day-old Arabidopsis seedlings resulted in the up-regulation of several known plant defense genes and markers of innate immunity, such as the transcription factors *WRKY29* and *WRKY33*, *PATHOGENESIS-RELATED* (*PR*) genes (*PR-1*, *PR-2*, and *PR-5*), and *FRK1*, all of which are also up-regulated during the onset of plant defenses against pathogens [29]. Two recent studies also showed a significant overlap between the genome-wide expression changes in plants treated with either hypoxia or a PAMP such as flg22 [30,31], with some of these overlaps being conserved, at least within the Brassicaceae family [30]. Genes commonly regulated by both hypoxia and flg22 largely showed the same directionality of gene expression change. These included genes with well-established roles in plant immunity and defense (e.g., *WRKY33* and *CPK28*) or genes with key roles in response to hypoxia (e.g., *HYPOXIA RESPONSIVE ERF 1* (*HRE1*), *HRE2*, *PHYTOGLOBIN 1* (*PGB1*) and *Alanine AMINOTRANSFERASE1* (*AlaAT1*)) [30]. Apart from a few regulators which have been experimentally shown to play roles in response to both hypoxia and defense (see below), in many cases, the dual role of many of these commonly differentially expressed genes remains to be established. Nevertheless, altogether, these observations suggest that some transcriptional regulators could play a “dual role” in both flooding/hypoxia response and during the onset of immune and defense responses.

This idea of “dual role” transcription factors is supported experimentally, as known regulators of hypoxia response have been shown to also play roles in immunity, while well-established transcriptional regulators of plant defenses against pathogens have subsequently been shown to also play roles in hypoxia response, thus linking the two transcriptional response programs. The ERF-VII transcription factors that act as master regulators of the hypoxia response also play a role in mediating plant responses to pathogens. For example, the increased susceptibility phenotype to *Plamodiophora brassiccae* of a *prt6* N-degron pathway mutant that constitutively accumulates ERF-VII transcription factors was rescued in a *prt6 erfVII* sextuple mutant [124]. In addition, the barley homolog of Arabidopsis *RAP2.2* (*HvRAP2.2*) was shown to activate the expression of immune marker genes such as *PR1* and confer increased resistance to the bacterial wilt pathogen *Ralstonia solanacearum* when it was overexpressed in barley [125]. Similarly, *Arabidopsis thaliana* plants overexpressing *RAP2.2* have increased resistance to the necrotrophic pathogen *Botrytis cinerea*, while *rap2.2* mutants are more susceptible to this pathogen [126]. Interestingly, this pathogen was shown to trigger local hypoxic niches during infection (see also below) [127], which could stabilize the ERF-VII transcription factors and perhaps enable their role in defense against *B. cinerea*. RAP2.3 has also been shown to mediate plant resistance to the necrotrophic bacterial pathogen *Pectobacterium carotovorum*, together with ORA59, a known regulator of JA and ethylene responses [128]. Indeed, a *rap2.3* mutant had increased susceptibility to *P. carotovorum*, similarly to an *ora59* mutant, in agreement with the protein/protein interactions between the two transcriptional regulators [128].

The ERF-VII transcription factors RAP2.2 and RAP2.12 up-regulate *RBOHD* expression under a range of abiotic stresses other than hypoxia (e.g., drought and high light), thus possibly linking the master regulators of hypoxia response to the transcriptional regulation of *RBOHD* [96]. In contrast, HRE1, another ERF-VII transcription factor, appears to repress *RBOHD* expression under hypoxia [129]. It is unclear why different sets of ERF-VII transcription factors might have opposing roles in *RBOHD* expression. Interestingly, under combined hypoxia_x_flg22 treatment of wild-type Arabidopsis seedlings, the repression of MAPK signaling and some of the transcriptional changes appeared to be mostly independent of ERF-VII function, as *erfVII* quintuple-mutant plants behaved similarly to the wild type [30].

Conversely, transcriptional regulators of plant immunity and defenses against pathogens can play roles in hypoxia response. WRKY33 is a well-known regulator of plant responses to pathogens and PAMPs (e.g., [130,131]) which has recently been shown to also contribute to submergence tolerance by up-regulating (together with WRKY12) the expression of *RAP2.2*. In agreement with this role, *wrky33* and *wrky12* mutant plants have decreased tolerance to submergence and to the subsequent reoxygenation period [132]. Notably, RAP2.2 also appears to act as a positive transcriptional regulator of *WRKY33*, thus potentially establishing a positive feedback loop between hypoxia response and defense against pathogens [132]. This is reminiscent of the activation of *WRKY33* by RAP2.3 in the context of plant responses to *B. cinerea* [120]. In addition, WRKY33 is also post-translationally regulated via its degradation mediated by the E3 ligase SUBMERGENCE RESISTANT 1 (SR1) upon reoxygenation. SR1 protein levels decrease during dark submergence, correlating with an increase in WRKY33 protein levels, while reoxygenation is associated with a concomitant increase in SR1 levels and a decrease in WRKY33 protein levels. The E3 ligase SR1 thus acts as a negative regulator of submergence response [133]. As discussed earlier, WRKY22 is also involved in both hypoxia and immunity [29]. In rice, *OsWRKY62* is induced by submergence or hypoxia treatments and regulates SA-dependent immunity genes, such as *PR1a* and a transcriptional regulator of the biosynthesis genes for defense-related secondary metabolites. *OsWRKY62* knock-down rice plants treated with hypoxia also show a reduced induction of specific hypoxia response genes, suggesting that *OsWRKY62* is also involved in hypoxia response [134].

Comparison of the genome-wide gene expression changes in wild-type Arabidopsis seedlings treated with either hypoxia or flg22 and with simultaneous hypoxia_x_flg22 revealed that the transcriptional response to the joint treatment had novel features not found in the transcriptional response to each of the individual treatments. This result suggests that combined treatment may involve new sets of transcriptional regulators compared to each of the individual treatments. Amongst these hypoxia_x_flg22-specific genes, enrichment of the WRKY, NAC, and bZIP transcription factors was found, suggesting that they may play particularly important roles under combined treatment [30].

### 3.5. Hormone Signaling in the Crosstalk between Hypoxia and Immunity

Phytohormone signaling pathways play a crucial role in plant defenses against pathogens and in hypoxia response. We refer the reader to these excellent reviews, which cover the role of phytotohormones in immunity and defense in depth [28,101,135,136], including the crosstalk between JA, SA, and ethylene signaling. In terms of hormonal signaling during flooding/hypoxia, we refer the readers to the comprehensive reviews [137,138,139]. Here, ethylene will be briefly discussed because of its predominant role in hypoxia response, while also interacting with SA and JA signaling in the context of plant immunity and defense [140]. Ethylene is a gaseous plant hormone whose production is induced after flg22 or pathogen recognition and plays particularly important roles upon infection with necrotrophic pathogens, together with JA signaling [140]. For example, ethylene synergizes the so-called ERF branch of JA signaling to activate defense-related genes such as *PLANT DEFENSIN 1.2* (*PDF1.2*), *ERF1*, and *ORA59* [141,142,143]. Ethylene has also been shown to be important for early PTI signaling events via the regulation of RBOHD and the transcriptional up-regulation of *FLS2* [144].

During flooding, ethylene is trapped within plant tissues and activates early acclimation to flooding and hypoxia [76,145,146] by (i) promoting the expression of *PGB1*, which, in turn, decreases NO levels, resulting in the stabilization of ERF-VII transcription factors [76], and (ii) up-regulating hypoxia response genes, including some involved in the regulation of ROS [146]. Furthermore, ethylene trapped in submerged plant tissues represses translation in general but at the same time enhances the translation of mRNAs coding for specific hypoxia response genes via the regulation of ETHYLENE-INSENSITIVE 2 (EIN2; an important positive regulator of ethylene signaling) and the serine/threonine kinase protein and sensor of nutrient starvation GENERAL CONTROL NONDEREPRESSIBLE 2 (GCN2), which phosphorylates eukaryotic initiation factor 2α (eIF2α) [147]. This study also showed that EIN2 modulates the ethylene/JA-mediated defense response during submergence, thus providing an additional link between hypoxia and defense responses [147]. Similarly to the up-regulation of *GCN2* under flooding/hypoxia, GCN2 activity in *A. thaliana* is also reported to be activated by bacterial infection [148]. For example, upon bacterial infection, Arabidopsis GCN2 phosphorylates eIF2α and activates the translation of *TL1-BINDING TRANSCRIPTION FACTOR 1* (*TBF1*), which, in turn, modulates pre-invasive and post-invasive immunity. Ethylene might also contribute to regulating the expression of other hypoxia response genes, such as *HYPOXIA-RESPONSIVE MODULATOR 1* (*HRM1*), whose expression depends on ETHYLENE-INSENSITIVE 3 (EIN3, an important regulator of ethylene response genes) and RAP2.2 [149].

## 4. The Role of Local/Physiological Hypoxic Niches during Pathogen Infection or during the Onset of Immune and Defense Responses

Although hypoxia is often thought of in the context of flooding, it can also occur naturally in plant tissues or at the site of pathogen infection (reviewed in [22]). Direct evidence for pathogen-induced hypoxic niches at the site of infection in Arabidopsis has been reported upon infection with the necrotrophic fungal pathogens *Botrytis cinerea* and *Alternaria brassicicola.* Local hypoxia was demonstrated using complementary approaches, including (i) a GUS reporter under the control of the *PLANT CYSTEINE OXIDASE 1* (*PCO1*) promoter, as this gene shows strong and specific induction under hypoxic conditions, and (ii) direct oxygen measurement, which indicated a decrease in oxygen from 18% in uninfected leaf tissue to 2% in infected leaves [127]. Additionally, this study identified the induced expression of several hypoxia-response genes, including *HRE2*. The formation of these hypoxic niches is thought to be the result of increased respiration in infected tissue which locally depletes oxygen [127], although it may also be a consequence of water-soaking lesions and lower oxygen diffusion. Additional direct evidence for local *in planta* hypoxic conditions was provided during infection by the hemibiotrophic fungal pathogen *Magnaporthe oryzae* [150]. In this study, the establishment of local hypoxia in rice tissue during infection by *M. oryzae* was established using a non-invasive oxygen sensing system, as well as an immunofluorescent chemical probe. The induced expression of known rice hypoxia response genes, such as *OsCIPK15* (*CALCINEURIN B LIKE (CBL) INTERACTING PROTEIN KINASE*), *OsHIGD2* (*HYPOXIA-INDUCED GENE DOMAIN 2*), and *ALCOHOL DEHYDROGENASE 2* (*ADH2*), also validated the occurrence of hypoxia during infection by *M. oryzae* [150].

Hypoxia also occurs in plant tissue infected with pathogens that trigger the formation of tumors. This includes the club root disease-causing pathogen *Plasmodiophora brassicae* [124] and the tumors formed during *Agrobacterium tumefaciens* infection [151]. In the presence of *P. brassicae*, induced expression of the key hypoxia marker genes *ALCOHOL DEHYDROGENASE 1* (*ADH1*), *PYRUVATE DECARBOXYLASE 1* (*PDC1*), and *PDC2* during secondary root infection was observed, indicating pathogen-induced localized oxygen deficiency [124]. Similar observations of hypoxia response gene induction were made for *A. tumefaciens* tumor formation [151]. Interestingly, in the case of both *P. brassicae* and *A. tumefaciens*, an *erfVII* quintuple mutant showed reduced susceptibility to the pathogens [124,151]. In sum, these observations indicate that both the pathogen and the host experience hypoxia during pathogenesis.

The formation of local hypoxic niches during infection is relevant to our understanding of plant immunity and/or pathogen virulence. However, many open questions remain, such as, for example, understanding whether these hypoxic microenvironments negatively impact the onset of plant immunity and plant defenses against pathogens or instead whether they promote specific mechanisms of pathogen virulence, as suggested by several studies in different plant species and with a range of pathogens [150,152,153]. Interestingly, flg22 treatment alone does not appear to trigger the formation of such hypoxic niches [30,127], suggesting that they are particularly relevant in the context of plant/pathogen interaction.

## 5. Flooding and Its Influence on the Soil Microbiome

Plants in nature live in association with a multitude of microorganisms. The rhizosphere comprises the soil microorganism populations that are biochemically affected by plant root exudates and includes microorganisms with commensal, pathogenic, or symbiotic relationships with plant hosts [154]. Increasingly, it is recognized that the microbiome can expand plants’ defensive capabilities, often influencing the outcome of plant/pathogen interactions by acting as a barrier to pathogen invasion through (i) direct microbe/microbe interactions; (ii) priming of the plant immune system; and (iii) controlling microorganism populations in the soil (e.g., through the production of antibiotics) (reviewed in [155,156,157,158,159,160,161]). However, soil microbial populations can be influenced by environmental factors such as flooding. Specifically, reduced oxygen availability decreases the diversity of the soil microbiome and favors the establishment of facultative and/or strict anaerobes [20,154,162,163,164,165,166]. For example, a study in a pot system using wheat as a model plant showed that flooding led to an increase in the number of bacterial anaerobic species, but also of pathogenic ones, while the population of beneficial bacteria decreased [164]. Similar results were found for fungal populations [20]. In addition, the scale of changes varied depending on the developmental stage, with younger plants being associated with a more variable microbiome [164]. We refer the reader to this extensive review on the topic [163], while this review focuses briefly on how flooding influences the rhizosphere.

The release of plant hormones and other chemical compounds from the roots (i.e., root exudates) can influence microbial growth and attract/repel microbes [167,168,169]. The so-called “cry for help” hypothesis provides a framework to explain and study how stress-induced changes in root exudates can provide chemical cues that attract beneficial microorganisms and contribute to minimizing stress-related damage [168]. Experiments with *Agropyron cristatum* (crested wheatgrass) treated with different abiotic stresses showed that root exudates are altered in response to flooding [170]. However, it remains to be determined how these exudate changes affect the rhizosphere. In addition, as part of their response to flooding, plants switch to anaerobic fermentation to regenerate NAD+ and maintain ATP production through glycolysis. This switch is accompanied by the production of ethanol, which can act as a chemical attractant for different root pathogens, as well as favoring the presence of ethanol-catabolizing microorganisms [162,171,172]. Phytohormones also influence the rhizosphere. Ethylene accumulates in plants and plays a particularly important role in the onset of plant responses to flooding [76]. Notably, ethylene also inhibits the colonization processes of some bacterial endophytes [173]. Despite the progress made in understanding the metabolomic changes in plants in response to hypoxia/flooding, the molecular mechanisms and the changes in exudate composition in response to flooding remain under-explored. How these changes contribute to shaping the soil microbiome is also still poorly understood.

Understanding how flooding influences the composition and function of microbial communities in the soil and more specifically in the rhizosphere still requires research. Similarly, how these microbial communities modulate plant responses to pathogen infection remains largely unknown and is an intriguing area of research that could contribute solutions to enhance plant resilience to pathogen infection during flooding and minimize yield losses.

## 6. Conclusions

The more extreme climate conditions and increased stress frequency and severity imposed on plants due to the effects of global climate change have highlighted the need to understand how plants withstand the complex and changing environment in which they live. The decade-long focus on plant responses to individual stresses has revealed key sensing mechanisms, signaling pathways, and both transcriptional and post-transcriptional regulatory mechanisms underpinning plants’ responses to a wide range of abiotic stresses. Similar discoveries were made over decades of work on plant/pathogen interactions, from the detection of pathogens to the onset of immune responses and the activation of defenses against different types of pathogens. However, studying single stressors in plants often overlooks the complexity of the molecular mechanisms that underpin the sensing and response to multiple stressors which adversely affect crop productivity. Many signaling pathways and regulators have been shown to play dual or even multifactorial roles in the regulation of plant responses to multiple (a)biotic stresses. However, studying plant responses to combined simultaneous stresses has also revealed that such treatments often trigger novel responses that cannot be predicted from the knowledge gained from individual stresses, thus pointing to gaps of knowledge in our ability to comprehend how plants integrate information from multiple stresses. So far, much of the focus has been on the combination of abiotic stresses, particularly those involving heat and drought. However, biotic/abiotic stress interactions are also increasingly studied. In this context, flooding and hypoxic conditions are particularly interesting, as hypoxia may be the result of flooding-associated environmental stress but may also occur in a physiological setting [174] depending on the tissue considered or, in some cases, in the context of plant infection by pathogens.

The few molecular and biochemical mechanisms identified for combined flooding/hypoxia and pathogen infection have originated from experiments with the model plant *Arabidopsis thaliana* in controlled conditions. In many cases, it remains to be determined whether the potential regulators of (combined) flooding/hypoxia and defense also act using similar mechanisms in crop species (either dicots or monocots). Some studies in rice have, for example, already revealed some differences [123]. In addition to using plant species other than Arabidopsis, a more holistic approach that includes consideration of the microbiome and small-molecule communication between plants and micro-organisms or between microorganisms in a flooded environment is also crucial. Interestingly, similarly to plants, transcriptomic data on *M. oryzae* under hypoxic conditions have shown that this fungal pathogen regulates the expression of a large number of its own genes in response to hypoxia, such as those involved in mycelial development, sterol, fatty acid, and heme biosynthesis, and redox processes [175]. Therefore, it will also be intriguing to investigate the role of these metabolic adaptations in fungal virulence and their adaptation in hypoxic conditions [153]. More generally, our limited knowledge about the microbiome in the rhizosphere and its functional relevance under combined flooding_x_pathogen stress needs to be addressed to develop meaningful applications that may contribute to mitigating crop losses resulting from global climate change. Similarly, the transition from hypoxia to reoxygenation in plants under combined stress is often overlooked and yet likely affects the mechanism of plants’ response to pathogens under combined stress conditions [24].

## Data Availability

No new data were created or analyzed in this study. Data sharing is not applicable to this article.

## References

[1] Atkinson N.J., Urwin P.E. (2012). The interaction of plant biotic and abiotic stresses: From genes to the field. J. Exp. Bot..

[2] Kissoudis C., van de Wiel C., Visser R.G., van der Linden G. (2014). Enhancing crop resilience to combined abiotic and biotic stress through the dissection of physiological and molecular crosstalk. Front. Plant Sci..

[3] Pandey P., Ramegowda V., Senthil-Kumar M. (2015). Shared and unique responses of plants to multiple individual stresses and stress combinations: Physiological and molecular mechanisms. Front. Plant Sci..

[4] Prasch C.M., Sonnewald U. (2013). Simultaneous application of heat, drought, and virus to Arabidopsis plants reveals significant shifts in signaling networks. Plant Physiol..

[5] Zhang H., Sonnewald U. (2017). Differences and commonalities of plant responses to single and combined stresses. Plant J..

[6] Zandalinas S.I., Casal J., Rouached H., Mittler R. (2024). Stress combination: From genes to ecosystems. Plant J..

[7] Suzuki N., Rivero R.M., Shulaev V., Blumwald E., Mittler R. (2014). Abiotic and biotic stress combinations. New Phytol..

[8] Tan Q.W., Lim P.K., Chen Z., Pasha A., Provart N., Arend M., Nikoloski Z., Mutwil M. (2023). Cross-stress gene expression atlas of Marchantia polymorpha reveals the hierarchy and regulatory principles of abiotic stress responses. Nat. Commun..

[9] Liu K., Harrison M.T., Yan H., Liu L., Meinke H., Hoogenboom G., Wang B., Peng B., Guan K., Jaegermeyr J. (2023). Silver lining to a climate crisis in multiple prospects for alleviating crop waterlogging under future climates. Nat. Commun..

[10] Kissoudis C., Sunarti S., van de Wiel C., Visser R.G., van der Linden C.G., Bai Y. (2016). Responses to combined abiotic and biotic stress in tomato are governed by stress intensity and resistance mechanism. J. Exp. Bot..

[11] Pastori G.M., Foyer C.H. (2002). Common components, networks, and pathways of cross-tolerance to stress. The central role of “redox” and abscisic acid-mediated controls. Plant Physiol..

[12] Roussin-Leveillee C., Rossi C.A.M., Castroverde C.D.M., Moffett P. (2024). The plant disease triangle facing climate change: A molecular perspective. Trends Plant Sci..

[13] Saijo Y., Loo E.P. (2020). Plant immunity in signal integration between biotic and abiotic stress responses. New Phytol..

[14] Hirabayashi Y., Mahendran R., Koirala S., Konoshima L., Yamazaki D., Watanabe S., Kim H., Kanae S. (2013). Global flood risk under climate change. Nat. Clim. Chang..

[15] Voesenek L.A., Bailey-Serres J. (2015). Flood adaptive traits and processes: An overview. New Phytol..

[16] FAO (2021). The Impact of Disasters and Crises on Agriculture and Food Security: 2021.

[17] Kim W., Iizumi T., Hosokawa N., Tanoue M., Hirabayashi Y. (2023). Flood impacts on global crop production: Advances and limitations. Environ. Res. Lett..

[18] Sasidharan R., Bailey-Serres J., Ashikari M., Atwell B.J., Colmer T.D., Fagerstedt K., Fukao T., Geigenberger P., Hebelstrup K.H., Hill R.D. (2017). Community recommendations on terminology and procedures used in flooding and low oxygen stress research. New Phytol..

[19] Setter T.L., Waters I. (2003). Review of prospects for germplasm improvement for waterlogging tolerance in wheat, barley and oats. Plant Soil.

[20] Francioli D., Cid G., Hajirezaei M.R., Kolb S. (2022). Response of the wheat mycobiota to flooding revealed substantial shifts towards plant pathogens. Front. Plant Sci..

[21] Salunkhe V.N., Gedam P., Pradhan A., Gaikwad B., Kale R., Gawande S. (2022). Concurrent waterlogging and anthracnose-twister disease in rainy-season onions (Allium cepa): Impact and management. Front. Microbiol..

[22] Loreti E., Perata P. (2020). The Many Facets of Hypoxia in Plants. Plants.

[23] Torres-Martínez L., Sánchez-Julia M., Kimbrough E., Hendrix T.C., Hendrix M., Day R.H., Krauss K.W., Van Bael S.A. (2020). Influence of soil microbiota on Taxodium distichum seedling performance during extreme flooding events. Plant Ecol..

[24] Yuan L.B., Chen M.X., Wang L.N., Sasidharan R., Voesenek L., Xiao S. (2023). Multi-stress resilience in plants recovering from submergence. Plant Biotechnol. J..

[25] Brazel A.J., Graciet E. (2023). Complexity of Abiotic Stress Stimuli: Mimicking Hypoxic Conditions Experimentally on the Basis of Naturally Occurring Environments. Methods Mol. Biol..

[26] Lee S.C., Mustroph A., Sasidharan R., Vashisht D., Pedersen O., Oosumi T., Voesenek L.A., Bailey-Serres J. (2011). Molecular characterization of the submergence response of the Arabidopsis thaliana ecotype Columbia. New Phytol..

[27] Felix G., Duran J.D., Volko S., Boller T. (1999). Plants have a sensitive perception system for the most conserved domain of bacterial flagellin. Plant J..

[28] Pieterse C.M., Van der Does D., Zamioudis C., Leon-Reyes A., Van Wees S.C. (2012). Hormonal modulation of plant immunity. Annu. Rev. Cell Dev. Biol..

[29] Hsu F.C., Chou M.Y., Chou S.J., Li Y.R., Peng H.P., Shih M.C. (2013). Submergence confers immunity mediated by the WRKY22 transcription factor in Arabidopsis. Plant Cell.

[30] Mooney B.C., Doorly C.M., Mantz M., García P., Huesgen P.F., Graciet E. (2024). Repression of pattern-triggered immune responses by hypoxia in Arabidopsis. Plant Physiol..

[31] Liu Z., Zhou Y., Liu Y., Qin A., Sun S., Liu H., Yang J., Hu M., Xie Y., Song X. Single-Cell RNA-Sequencing Reveals the Generation of New Immune Response-Induced Cells with Novel Role in *Arabidopsis thaliana* Response to the Bacterial Flagellin Epitope flg22. https://papers.ssrn.com/sol3/papers.cfm?abstract_id=4548674.

[32] Tang H., Liu H. (2021). Roles of single gene in plant hypoxia and pathogen responses. Plant Signal. Behav..

[33] Jones J.D., Dangl J.L. (2006). The plant immune system. Nature.

[34] Jones J.D.G., Staskawicz B.J., Dangl J.L. (2024). The plant immune system: From discovery to deployment. Cell.

[35] Rhodes J., Zipfel C., Jones J.D.G., Ngou B.P.M. (2022). Concerted actions of PRR- and NLR-mediated immunity. Essays Biochem..

[36] Ngou B.P.M., Ding P., Jones J.D.G. (2022). Thirty years of resistance: Zig-zag through the plant immune system. Plant Cell.

[37] Chinchilla D., Bauer Z., Regenass M., Boller T., Felix G. (2006). The Arabidopsis receptor kinase FLS2 binds flg22 and determines the specificity of flagellin perception. Plant Cell.

[38] Gomez-Gomez L., Boller T. (2000). FLS2: An LRR receptor-like kinase involved in the perception of the bacterial elicitor flagellin in Arabidopsis. Mol. Cell.

[39] Sun Y., Li L., Macho A.P., Han Z., Hu Z., Zipfel C., Zhou J.M., Chai J. (2013). Structural basis for flg22-induced activation of the Arabidopsis FLS2-BAK1 immune complex. Science.

[40] Chinchilla D., Zipfel C., Robatzek S., Kemmerling B., Nurnberger T., Jones J.D., Felix G., Boller T. (2007). A flagellin-induced complex of the receptor FLS2 and BAK1 initiates plant defence. Nature.

[41] Heese A., Hann D.R., Gimenez-Ibanez S., Jones A.M.E., He K., Li J., Schroeder J.I., Peck S.C., Rathjen J.P. (2007). The receptor-like kinase SERK3/BAK1 is a central regulator of innate immunity in plants. Proc. Natl. Acad. Sci. USA.

[42] Macho A.P., Zipfel C. (2014). Plant PRRs and the activation of innate immune signaling. Mol. Cell.

[43] Li L., Li M., Yu L., Zhou Z., Liang X., Liu Z., Cai G., Gao L., Zhang X., Wang Y. (2014). The FLS2-associated kinase BIK1 directly phosphorylates the NADPH oxidase RbohD to control plant immunity. Cell Host Microbe.

[44] Lu D., Wu S., Gao X., Zhang Y., Shan L., He P. (2010). A receptor-like cytoplasmic kinase, BIK1, associates with a flagellin receptor complex to initiate plant innate immunity. Proc. Natl. Acad. Sci. USA.

[45] Giuntoli B., Perata P. (2018). Group VII Ethylene Response Factors in Arabidopsis: Regulation and Physiological Roles. Plant Physiol..

[46] Licausi F., van Dongen J.T., Giuntoli B., Novi G., Santaniello A., Geigenberger P., Perata P. (2010). HRE1 and HRE2, two hypoxia-inducible ethylene response factors, affect anaerobic responses in Arabidopsis thaliana. Plant J..

[47] Gibbs D.J., Lee S.C., Isa N.M., Gramuglia S., Fukao T., Bassel G.W., Correia C.S., Corbineau F., Theodoulou F.L., Bailey-Serres J. (2011). Homeostatic response to hypoxia is regulated by the N-end rule pathway in plants. Nature.

[48] Licausi F., Kosmacz M., Weits D.A., Giuntoli B., Giorgi F.M., Voesenek L.A., Perata P., van Dongen J.T. (2011). Oxygen sensing in plants is mediated by an N-end rule pathway for protein destabilization. Nature.

[49] Weits D.A., Giuntoli B., Kosmacz M., Parlanti S., Hubberten H.M., Riegler H., Hoefgen R., Perata P., van Dongen J.T., Licausi F. (2014). Plant cysteine oxidases control the oxygen-dependent branch of the N-end-rule pathway. Nat. Commun..

[50] White M.D., Kamps J., East S., Taylor Kearney L.J., Flashman E. (2018). The plant cysteine oxidases from Arabidopsis thaliana are kinetically tailored to act as oxygen sensors. J. Biol. Chem..

[51] White M.D., Klecker M., Hopkinson R.J., Weits D.A., Mueller C., Naumann C., O’Neill R., Wickens J., Yang J., Brooks-Bartlett J.C. (2017). Plant cysteine oxidases are dioxygenases that directly enable arginyl transferase-catalysed arginylation of N-end rule targets. Nat. Commun..

[52] Lee T.A., Bailey-Serres J. (2019). Integrative Analysis from the Epigenome to Translatome Uncovers Patterns of Dominant Nuclear Regulation during Transient Stress. Plant Cell.

[53] Gasch P., Fundinger M., Muller J.T., Lee T., Bailey-Serres J., Mustroph A. (2016). Redundant ERF-VII Transcription Factors Bind to an Evolutionarily Conserved cis-Motif to Regulate Hypoxia-Responsive Gene Expression in Arabidopsis. Plant Cell.

[54] Mustroph A., Lee S.C., Oosumi T., Zanetti M.E., Yang H., Ma K., Yaghoubi-Masihi A., Fukao T., Bailey-Serres J. (2010). Cross-kingdom comparison of transcriptomic adjustments to low-oxygen stress highlights conserved and plant-specific responses. Plant Physiol..

[55] Perata P., Armstrong W., Voesenek L.A. (2011). Plants and flooding stress. New Phytol..

[56] Loreti E., Valeri M.C., Novi G., Perata P. (2018). Gene Regulation and Survival under Hypoxia Requires Starch Availability and Metabolism. Plant Physiol..

[57] Loreti E., van Veen H., Perata P. (2016). Plant responses to flooding stress. Curr. Opin. Plant Biol..

[58] Jeworutzki E., Roelfsema M.R., Anschutz U., Krol E., Elzenga J.T., Felix G., Boller T., Hedrich R., Becker D. (2010). Early signaling through the Arabidopsis pattern recognition receptors FLS2 and EFR involves Ca-associated opening of plasma membrane anion channels. Plant J..

[59] Ranf S., Eschen-Lippold L., Pecher P., Lee J., Scheel D. (2011). Interplay between calcium signalling and early signalling elements during defence responses to microbe- or damage-associated molecular patterns. Plant J..

[60] Marcec M.J., Tanaka K. (2021). Crosstalk between Calcium and ROS Signaling during Flg22-Triggered Immune Response in Arabidopsis Leaves. Plants.

[61] Sedbrook J.C., Kronebusch P.J., Borisy G.G., Trewavas A.J., Masson P.H. (1996). Transgenic AEQUORIN reveals organ-specific cytosolic Ca2+ responses to anoxia and Arabidopsis thaliana seedlings. Plant Physiol..

[62] Subbaiah C.C., Bush D.S., Sachs M.M. (1998). Mitochondrial contribution to the anoxic Ca2+ signal in maize suspension-cultured cells. Plant Physiol..

[63] Subbaiah C.C., Bush D.S., Sachs M.M. (1994). Elevation of cytosolic calcium precedes anoxic gene expression in maize suspension-cultured cells. Plant Cell.

[64] Tian W., Wang C., Gao Q., Li L., Luan S. (2020). Calcium spikes, waves and oscillations in plant development and biotic interactions. Nat. Plants.

[65] Yemelyanov V.V., Shishova M.F., Chirkova T.V., Lindberg S.M. (2011). Anoxia-induced elevation of cytosolic Ca2+ concentration depends on different Ca^2+^ sources in rice and wheat protoplasts. Planta.

[66] Bakshi A., Choi W.G., Kim S.H., Gilroy S. (2023). The vacuolar Ca(2+) transporter CATION EXCHANGER 2 regulates cytosolic calcium homeostasis, hypoxic signaling, and response to flooding in Arabidopsis thaliana. New Phytol..

[67] Wagner S., Steinbeck J., Fuchs P., Lichtenauer S., Elsasser M., Schippers J.H.M., Nietzel T., Ruberti C., Van Aken O., Meyer A.J. (2019). Multiparametric real-time sensing of cytosolic physiology links hypoxia responses to mitochondrial electron transport. New Phytol..

[68] Delledonne M., Xia Y., Dixon R.A., Lamb C. (1998). Nitric oxide functions as a signal in plant disease resistance. Nature.

[69] Durner J., Wendehenne D., Klessig D.F. (1998). Defense gene induction in tobacco by nitric oxide, cyclic GMP, and cyclic ADP-ribose. Proc. Natl. Acad. Sci. USA.

[70] Modolo L.V., Augusto O., Almeida I.M., Magalhaes J.R., Salgado I. (2005). Nitrite as the major source of nitric oxide production by Arabidopsis thaliana in response to Pseudomonas syringae. FEBS Lett..

[71] Mugnai S., Azzarello E., Baluska F., Mancuso S. (2012). Local root apex hypoxia induces NO-mediated hypoxic acclimation of the entire root. Plant Cell Physiol..

[72] Gibbs D.J., Md Isa N., Movahedi M., Lozano-Juste J., Mendiondo G.M., Berckhan S., Marin-de la Rosa N., Vicente Conde J., Sousa Correia C., Pearce S.P. (2014). Nitric oxide sensing in plants is mediated by proteolytic control of group VII ERF transcription factors. Mol. Cell.

[73] Gautier C., van Faassen E., Mikula I., Martasek P., Slama-Schwok A. (2006). Endothelial nitric oxide synthase reduces nitrite anions to NO under anoxia. Biochem. Biophys. Res. Commun..

[74] Mikula I., Durocher S., Martasek P., Mutus B., Slama-Schwok A. (2009). Isoform-specific differences in the nitrite reductase activity of nitric oxide synthases under hypoxia. Biochem. J..

[75] Vanin A.F., Bevers L.M., Slama-Schwok A., van Faassen E.E. (2007). Nitric oxide synthase reduces nitrite to NO under anoxia. Cell. Mol. Life Sci..

[76] Hartman S., Liu Z., van Veen H., Vicente J., Reinen E., Martopawiro S., Zhang H., van Dongen N., Bosman F., Bassel G.W. (2019). Ethylene-mediated nitric oxide depletion pre-adapts plants to hypoxia stress. Nat. Commun..

[77] Choudhury F.K., Rivero R.M., Blumwald E., Mittler R. (2017). Reactive oxygen species, abiotic stress and stress combination. Plant J..

[78] Martinez Rivas F.J., Fernie A.R., Aarabi F. (2024). Roles and regulation of the RBOHD enzyme in initiating ROS-mediated systemic signaling during biotic and abiotic stress. Plant Stress.

[79] Castro B., Citterico M., Kimura S., Stevens D.M., Wrzaczek M., Coaker G. (2021). Stress-induced reactive oxygen species compartmentalization, perception and signalling. Nat. Plants.

[80] O’Brien J.A., Daudi A., Finch P., Butt V.S., Whitelegge J.P., Souda P., Ausubel F.M., Bolwell G.P. (2012). A peroxidase-dependent apoplastic oxidative burst in cultured Arabidopsis cells functions in MAMP-elicited defense. Plant Physiol..

[81] Vanacker H., Carver T.L., Foyer C.H. (1998). Pathogen-induced changes in the antioxidant status of the apoplast in barley leaves. Plant Physiol..

[82] Bienert G.P., Moller A.L., Kristiansen K.A., Schulz A., Moller I.M., Schjoerring J.K., Jahn T.P. (2007). Specific aquaporins facilitate the diffusion of hydrogen peroxide across membranes. J. Biol. Chem..

[83] Bienert G.P., Schjoerring J.K., Jahn T.P. (2006). Membrane transport of hydrogen peroxide. Biochim. Biophys. Acta.

[84] Jang J.Y., Rhee J.Y., Chung G.C., Kang H. (2012). Aquaporin as a membrane transporter of hydrogen peroxide in plant response to stresses. Plant Signal. Behav..

[85] Dynowski M., Schaaf G., Loque D., Moran O., Ludewig U. (2008). Plant plasma membrane water channels conduct the signalling molecule H_2_O_2_. Biochem. J..

[86] Kadota Y., Sklenar J., Derbyshire P., Stransfeld L., Asai S., Ntoukakis V., Jones J.D., Shirasu K., Menke F., Jones A. (2014). Direct regulation of the NADPH oxidase RBOHD by the PRR-associated kinase BIK1 during plant immunity. Mol. Cell.

[87] Dubiella U., Seybold H., Durian G., Komander E., Lassig R., Witte C.P., Schulze W.X., Romeis T. (2013). Calcium-dependent protein kinase/NADPH oxidase activation circuit is required for rapid defense signal propagation. Proc. Natl. Acad. Sci. USA.

[88] Rentel M.C., Knight M.R. (2004). Oxidative stress-induced calcium signaling in Arabidopsis. Plant Physiol..

[89] Boudsocq M., Willmann M.R., McCormack M., Lee H., Shan L., He P., Bush J., Cheng S.-H., Sheen J. (2010). Differential innate immune signalling via Ca^2+^ sensor protein kinases. Nature.

[90] Ogasawara Y., Kaya H., Hiraoka G., Yumoto F., Kimura S., Kadota Y., Hishinuma H., Senzaki E., Yamagoe S., Nagata K. (2008). Synergistic activation of the Arabidopsis NADPH oxidase AtrbohD by Ca2+ and phosphorylation. J. Biol. Chem..

[91] Cui B., Pan Q., Cui W., Wang Y., Loake V.I.P., Yuan S., Liu F., Loake G.J. (2024). S-nitrosylation of a receptor-like cytoplasmic kinase regulates plant immunity. Sci. Adv..

[92] Yun B.W., Feechan A., Yin M., Saidi N.B., Le Bihan T., Yu M., Moore J.W., Kang J.G., Kwon E., Spoel S.H. (2011). S-nitrosylation of NADPH oxidase regulates cell death in plant immunity. Nature.

[93] Lee D., Lal N.K., Lin Z.D., Ma S., Liu J., Castro B., Toruno T., Dinesh-Kumar S.P., Coaker G. (2020). Regulation of reactive oxygen species during plant immunity through phosphorylation and ubiquitination of RBOHD. Nat. Commun..

[94] Hong C.P., Wang M.C., Yang C.Y. (2020). NADPH Oxidase RbohD and Ethylene Signaling are Involved in Modulating Seedling Growth and Survival Under Submergence Stress. Plants.

[95] Yang C.-Y., Hong C.P. (2015). The NADPH oxidase Rboh D is involved in primary hypoxia signalling and modulates expression of hypoxia-inducible genes under hypoxic stress. Environ. Exp. Bot..

[96] Yao Y., He R.J., Xie Q.L., Zhao X.H., Deng X.M., He J.B., Song L., He J., Marchant A., Chen X.Y. (2017). ETHYLENE RESPONSE FACTOR 74 (ERF74) plays an essential role in controlling a respiratory burst oxidase homolog D (RbohD)-dependent mechanism in response to different stresses in Arabidopsis. New Phytol..

[97] Pucciariello C., Parlanti S., Banti V., Novi G., Perata P. (2012). Reactive oxygen species-driven transcription in Arabidopsis under oxygen deprivation. Plant Physiol..

[98] Liu B., Sun L., Ma L., Hao F.S. (2017). Both AtrbohD and AtrbohF are essential for mediating responses to oxygen deficiency in Arabidopsis. Plant Cell Rep..

[99] Pelaez-Vico M.A., Tukuli A., Singh P., Mendoza-Cozatl D.G., Joshi T., Mittler R. (2023). Rapid systemic responses of Arabidopsis to waterlogging stress. Plant Physiol..

[100] Evans M.J., Choi W.G., Gilroy S., Morris R.J. (2016). A ROS-Assisted Calcium Wave Dependent on the AtRBOHD NADPH Oxidase and TPC1 Cation Channel Propagates the Systemic Response to Salt Stress. Plant Physiol..

[101] Gilroy E., Breen S. (2022). Interplay between phytohormone signalling pathways in plant defence—Other than salicylic acid and jasmonic acid. Essays Biochem..

[102] Yu W.W., Chen Q.F., Liao K., Zhou D.M., Yang Y.C., He M., Yu L.J., Guo D.Y., Xiao S., Xie R.H. (2024). The calcium-dependent protein kinase CPK16 regulates hypoxia-induced ROS production by phosphorylating the NADPH oxidase RBOHD in Arabidopsis. Plant Cell.

[103] Gonzali S., Loreti E., Cardarelli F., Novi G., Parlanti S., Pucciariello C., Bassolino L., Banti V., Licausi F., Perata P. (2015). Universal stress protein HRU1 mediates ROS homeostasis under anoxia. Nat. Plants.

[104] Baxter-Burrell A., Yang Z., Springer P.S., Bailey-Serres J. (2002). RopGAP4-dependent Rop GTPase rheostat control of Arabidopsis oxygen deprivation tolerance. Science.

[105] Sun L., Ma L., He S., Hao F. (2018). AtrbohD functions downstream of ROP2 and positively regulates waterlogging response in Arabidopsis. Plant Signal. Behav..

[106] Miller G., Schlauch K., Tam R., Cortes D., Torres M.A., Shulaev V., Dangl J.L., Mittler R. (2009). The plant NADPH oxidase RBOHD mediates rapid systemic signaling in response to diverse stimuli. Sci. Signal..

[107] Gilroy S., Suzuki N., Miller G., Choi W.G., Toyota M., Devireddy A.R., Mittler R. (2014). A tidal wave of signals: Calcium and ROS at the forefront of rapid systemic signaling. Trends Plant Sci..

[108] Fichman Y., Mittler R. (2020). Rapid systemic signaling during abiotic and biotic stresses: Is the ROS wave master of all trades?. Plant J..

[109] Pelaez-Vico M.A., Fichman Y., Zandalinas S.I., Foyer C.H., Mittler R. (2024). ROS are universal cell-to-cell stress signals. Curr. Opin. Plant Biol..

[110] Zandalinas S.I., Fichman Y., Devireddy A.R., Sengupta S., Azad R.K., Mittler R. (2020). Systemic signaling during abiotic stress combination in plants. Proc. Natl. Acad. Sci. USA.

[111] Asai T., Tena G., Plotnikova J., Willmann M.R., Chiu W.L., Gomez-Gomez L., Boller T., Ausubel F.M., Sheen J. (2002). MAP kinase signalling cascade in Arabidopsis innate immunity. Nature.

[112] Colcombet J., Hirt H. (2008). Arabidopsis MAPKs: A complex signalling network involved in multiple biological processes. Biochem. J..

[113] Rodriguez M.C., Petersen M., Mundy J. (2010). Mitogen-activated protein kinase signaling in plants. Annu. Rev. Plant Biol..

[114] Wu G., Wang W. (2024). Recent advances in understanding the role of two mitogen-activated protein kinase cascades in plant immunity. J. Exp. Bot..

[115] Chang R., Jang C.J., Branco-Price C., Nghiem P., Bailey-Serres J. (2012). Transient MPK6 activation in response to oxygen deprivation and reoxygenation is mediated by mitochondria and aids seedling survival in Arabidopsis. Plant Mol. Biol..

[116] Mao G., Meng X., Liu Y., Zheng Z., Chen Z., Zhang S. (2011). Phosphorylation of a WRKY transcription factor by two pathogen-responsive MAPKs drives phytoalexin biosynthesis in Arabidopsis. Plant Cell.

[117] Menke F.L., van Pelt J.A., Pieterse C.M., Klessig D.F. (2004). Silencing of the mitogen-activated protein kinase MPK6 compromises disease resistance in Arabidopsis. Plant Cell.

[118] Zhou Y., Zhou D.M., Yu W.W., Shi L.L., Zhang Y., Lai Y.X., Huang L.P., Qi H., Chen Q.F., Yao N. (2022). Phosphatidic acid modulates MPK3- and MPK6-mediated hypoxia signaling in Arabidopsis. Plant Cell.

[119] Zhou J., Wang X., He Y., Sang T., Wang P., Dai S., Zhang S., Meng X. (2020). Differential Phosphorylation of the Transcription Factor WRKY33 by the Protein Kinases CPK5/CPK6 and MPK3/MPK6 Cooperatively Regulates Camalexin Biosynthesis in Arabidopsis. Plant Cell.

[120] Li Y., Liu K., Tong G., Xi C., Liu J., Zhao H., Wang Y., Ren D., Han S. (2022). MPK3/MPK6-mediated phosphorylation of ERF72 positively regulates resistance to Botrytis cinerea through directly and indirectly activating the transcription of camalexin biosynthesis enzymes. J. Exp. Bot..

[121] Sorensson C., Lenman M., Veide-Vilg J., Schopper S., Ljungdahl T., Grotli M., Tamas M.J., Peck S.C., Andreasson E. (2012). Determination of primary sequence specificity of Arabidopsis MAPKs MPK3 and MPK6 leads to identification of new substrates. Biochem. J..

[122] Cho H.Y., Wen T.N., Wang Y.T., Shih M.C. (2016). Quantitative phosphoproteomics of protein kinase SnRK1 regulated protein phosphorylation in Arabidopsis under submergence. J. Exp. Bot..

[123] Singh P., Sinha A.K. (2016). A Positive Feedback Loop Governed by SUB1A1 Interaction with MITOGEN-ACTIVATED PROTEIN KINASE3 Imparts Submergence Tolerance in Rice. Plant Cell.

[124] Gravot A., Richard G., Lime T., Lemarie S., Jubault M., Lariagon C., Lemoine J., Vicente J., Robert-Seilaniantz A., Holdsworth M.J. (2016). Hypoxia response in Arabidopsis roots infected by Plasmodiophora brassicae supports the development of clubroot. BMC Plant Biol..

[125] Jung J., Won S.Y., Suh S.C., Kim H., Wing R., Jeong Y., Hwang I., Kim M. (2007). The barley ERF-type transcription factor HvRAF confers enhanced pathogen resistance and salt tolerance in Arabidopsis. Planta.

[126] Zhao Y., Wei T., Yin K.Q., Chen Z., Gu H., Qu L.J., Qin G. (2012). Arabidopsis RAP2.2 plays an important role in plant resistance to Botrytis cinerea and ethylene responses. New Phytol..

[127] Valeri M.C., Novi G., Weits D.A., Mensuali A., Perata P., Loreti E. (2021). Botrytis cinerea induces local hypoxia in Arabidopsis leaves. New Phytol..

[128] Kim N.Y., Jang Y.J., Park O.K. (2018). AP2/ERF Family Transcription Factors ORA59 and RAP2.3 Interact in the Nucleus and Function Together in Ethylene Responses. Front. Plant Sci..

[129] Yang C.Y., Huang Y.C., Ou S.L. (2017). ERF73/HRE1 is involved in H(2)O(2) production via hypoxia-inducible Rboh gene expression in hypoxia signaling. Protoplasma.

[130] Birkenbihl R.P., Kracher B., Roccaro M., Somssich I.E. (2017). Induced Genome-Wide Binding of Three Arabidopsis WRKY Transcription Factors during Early MAMP-Triggered Immunity. Plant Cell.

[131] Birkenbihl R.P., Diezel C., Somssich I.E. (2012). Arabidopsis WRKY33 is a key transcriptional regulator of hormonal and metabolic responses toward Botrytis cinerea infection. Plant Physiol..

[132] Tang H., Bi H., Liu B., Lou S., Song Y., Tong S., Chen N., Jiang Y., Liu J., Liu H. (2021). WRKY33 interacts with WRKY12 protein to up-regulate RAP2.2 during submergence induced hypoxia response in Arabidopsis thaliana. New Phytol..

[133] Liu B., Jiang Y., Tang H., Tong S., Lou S., Shao C., Zhang J., Song Y., Chen N., Bi H. (2021). The ubiquitin E3 ligase SR1 modulates the submergence response by degrading phosphorylated WRKY33 in Arabidopsis. Plant Cell.

[134] Fukushima S., Mori M.M., Sugano S., Takatsuji H. (2016). Transcription Factor WRKY62 Plays a Role in Pathogen Defense and Hypoxia-Responsive Gene Expression in Rice. Plant Cell Physiol..

[135] Berens M.L., Berry H.M., Mine A., Argueso C.T., Tsuda K. (2017). Evolution of Hormone Signaling Networks in Plant Defense. Annu. Rev. Phytopathol..

[136] Aerts N., Pereira Mendes M., Van Wees S.C.M. (2021). Multiple levels of crosstalk in hormone networks regulating plant defense. Plant J..

[137] Wang X., Komatsu S. (2022). The Role of Phytohormones in Plant Response to Flooding. Int. J. Mol. Sci..

[138] Leon J., Castillo M.C., Gayubas B. (2021). The hypoxia-reoxygenation stress in plants. J. Exp. Bot..

[139] Bailey-Serres J., Voesenek L.A. (2008). Flooding stress: Acclimations and genetic diversity. Annu. Rev. Plant Biol..

[140] Broekgaarden C., Caarls L., Vos I.A., Pieterse C.M., Van Wees S.C. (2015). Ethylene: Traffic Controller on Hormonal Crossroads to Defense. Plant Physiol..

[141] Berrocal-Lobo M., Molina A., Solano R. (2002). Constitutive expression of ETHYLENE-RESPONSE-FACTOR1 in Arabidopsis confers resistance to several necrotrophic fungi. Plant J..

[142] Lorenzo O., Piqueras R., Sanchez-Serrano J.J., Solano R. (2003). ETHYLENE RESPONSE FACTOR1 integrates signals from ethylene and jasmonate pathways in plant defense. Plant Cell.

[143] Zhu Z., An F., Feng Y., Li P., Xue L., Jiang Z., Kim J.M., To T.K., Li W., Zhang X. (2011). Derepression of ethylene-stabilized transcription factors (EIN3/EIL1) mediates jasmonate and ethylene signaling synergy in Arabidopsis. Proc. Natl. Acad. Sci. USA.

[144] Mersmann S., Bourdais G., Rietz S., Robatzek S. (2010). Ethylene Signaling Regulates Accumulation of the FLS2 Receptor and Is Required for the Oxidative Burst Contributing to Plant Immunity. Plant Physiol..

[145] Maric A., Hartman S. (2022). Ethylene controls translational gatekeeping to overcome flooding stress in plants. EMBO J..

[146] Liu Z., Hartman S., van Veen H., Zhang H., Leeggangers H., Martopawiro S., Bosman F., de Deugd F., Su P., Hummel M. (2022). Ethylene augments root hypoxia tolerance via growth cessation and reactive oxygen species amelioration. Plant Physiol..

[147] Cho H.Y., Chou M.Y., Ho H.Y., Chen W.C., Shih M.C. (2022). Ethylene modulates translation dynamics in Arabidopsis under submergence via GCN2 and EIN2. Sci. Adv..

[148] Liu X., TAfrin K.M. (2019). Pajerowska-Mukhtar, Arabidopsis GCN2 kinase contributes to ABA homeostasis and stomatal immunity. Commun. Biol..

[149] Tsai K.J., Suen D.F., Shih M.C. (2023). Hypoxia response protein HRM1 modulates the activity of mitochondrial electron transport chain in Arabidopsis under hypoxic stress. New Phytol..

[150] Chung H., Kim S., Kim K.T., Hwang B.G., Kim H.J., Lee S.J., Lee Y.H. (2019). A novel approach to investigate hypoxic microenvironment during rice colonization by Magnaporthe oryzae. Environ. Microbiol..

[151] Kerpen L., Niccolini L., Licausi F., van Dongen J.T., Weits D.A. (2019). Hypoxic Conditions in Crown Galls Induce Plant Anaerobic Responses That Support Tumor Proliferation. Front. Plant Sci..

[152] Lisicka W., Fikowicz-Krosko J., Jafra S., Narajczyk M., Czaplewska P., Czajkowski R. (2018). Oxygen Availability Influences Expression of Dickeya solani Genes Associated With Virulence in Potato (*Solanum tuberosum* L.) and Chicory (*Cichorium intybus* L.). Front. Plant Sci..

[153] Chung H., Lee Y.H. (2020). Hypoxia: A Double-Edged Sword During Fungal Pathogenesis?. Front. Microbiol..

[154] Hartman K., Tringe S.G. (2019). Interactions between plants and soil shaping the root microbiome under abiotic stress. Biochem. J..

[155] Hacquard S., Spaepen S., Garrido-Oter R., Schulze-Lefert P. (2017). Interplay Between Innate Immunity and the Plant Microbiota. Annu. Rev. Phytopathol..

[156] Teixeira P.J.P., Colaianni N.R., Fitzpatrick C.R., Dangl J.L. (2019). Beyond pathogens: Microbiota interactions with the plant immune system. Curr. Opin. Microbiol..

[157] Vannier N., Agler M., Hacquard S. (2019). Microbiota-mediated disease resistance in plants. PLoS Pathog..

[158] Jian Y., Gong D., Wang Z., Liu L., He J., Han X., Tsuda K. (2024). How plants manage pathogen infection. EMBO Rep..

[159] Liu S., Tao C., Zhang L., Wang Z., Xiong W., Xiang D., Sheng O., Wang J., Li R., Shen Z. (2023). Plant pathogen resistance is mediated by recruitment of specific rhizosphere fungi. ISME J..

[160] Ma K.W., Niu Y., Jia Y., Ordon J., Copeland C., Emonet A., Geldner N., Guan R., Stolze S.C., Nakagami H. (2021). Coordination of microbe-host homeostasis by crosstalk with plant innate immunity. Nat. Plants.

[161] Pereira L.B., Thomazella D.P.T., Teixeira P. (2023). Plant-microbiome crosstalk and disease development. Curr. Opin. Plant Biol..

[162] Graff A., Conrad R. (2005). Impact of flooding on soil bacterial communities associated with poplar (*Populus* sp.) trees. FEMS Microbiol. Ecol..

[163] Martinez-Arias C., Witzell J., Solla A., Martin J.A., Rodriguez-Calcerrada J. (2022). Beneficial and pathogenic plant-microbe interactions during flooding stress. Plant Cell Environ..

[164] Francioli D., Cid G., Kanukollu S., Ulrich A., Hajirezaei M.R., Kolb S. (2021). Flooding Causes Dramatic Compositional Shifts and Depletion of Putative Beneficial Bacteria on the Spring Wheat Microbiota. Front. Microbiol..

[165] Abdul Rahman N.S.N., Abdul Hamid N.W., Nadarajah K. (2021). Effects of Abiotic Stress on Soil Microbiome. Int. J. Mol. Sci..

[166] Chialva M., Ghignone S., Cozzi P., Lazzari B., Bonfante P., Abbruscato P., Lumini E. (2020). Water management and phenology influence the root-associated rice field microbiota. FEMS Microbiol. Ecol..

[167] Bulgarelli D., Rott M., Schlaeppi K., Ver Loren van Themaat E., Ahmadinejad N., Assenza F., Rauf P., Huettel B., Reinhardt R., Schmelzer E. (2012). Revealing structure and assembly cues for Arabidopsis root-inhabiting bacterial microbiota. Nature.

[168] Rolfe S.A., Griffiths J., Ton J. (2019). Crying out for help with root exudates: Adaptive mechanisms by which stressed plants assemble health-promoting soil microbiomes. Curr. Opin. Microbiol..

[169] Meng H., Yan Z., Li X. (2022). Effects of exogenous organic acids and flooding on root exudates, rhizosphere bacterial community structure, and iron plaque formation in Kandelia obovata seedlings. Sci. Total Environ..

[170] Henry A., Doucette W., Norton J., Bugbee B. (2007). Changes in crested wheatgrass root exudation caused by flood, drought, and nutrient stress. J. Environ. Qual..

[171] Cameron J.N., Carlile M.J. (1978). Fatty acids, aldehydes and alcohols as attractants for zoospores of Phytophthora palmivora. Nature.

[172] Smucker A.J.M., Erickson A.E. (1987). Anaerobic stimulation of root exudates and disease of peas. Plant Soil.

[173] Iniguez A.L., Dong Y., Carter H.D., Ahmer B.M., Stone J.M., Triplett E.W. (2005). Regulation of enteric endophytic bacterial colonization by plant defenses. Mol. Plant-Microbe Interact. MPMI.

[174] Weits D.A., Kunkowska A.B., Kamps N.C.W., Portz K.M.S., Packbier N.K., Nemec Venza Z., Gaillochet C., Lohmann J.U., Pedersen O., van Dongen J.T. (2019). An apical hypoxic niche sets the pace of shoot meristem activity. Nature.

[175] Choi J., Chung H., Lee G.W., Koh S.K., Chae S.K., Lee Y.H. (2015). Genome-Wide Analysis of Hypoxia-Responsive Genes in the Rice Blast Fungus, Magnaporthe oryzae. PLoS ONE.

